# The Impact of Exclusion from Close Versus Distant Relationships on Inhibitory Control: An ERP Study

**DOI:** 10.3390/bs15101305

**Published:** 2025-09-24

**Authors:** Pengcheng Zhang, Xiangping Gao, Zhizhuan Li, Tongtong Xin

**Affiliations:** 1 Department of Psychology, School of Education, Zhejiang International Studies University, 299, Liuhe Road, Xihu, Hangzhou 310023, China; 2 Department of Psychology, Shanghai Normal University, Shanghai 200233, China; 3 Department of Psychology, Fuyang Normal University, Fuyang 236041, China

**Keywords:** social exclusion, close and distant relationships, inhibitory control, ERP

## Abstract

Previous research has not fully addressed the distinction between different sources of exclusion, focusing predominantly on how being excluded by strangers affects inhibitory control. To address this gap, this study employs a Go/Nogo task to examine how exclusion by individuals with varying degrees of social proximity (close vs. distant) affects inhibitory control. The results revealed that exclusion by both friends (*p* = 0.018) and strangers (*p* = 0.001) elicited significantly greater N270 amplitudes compared to inclusion by others. Conversely, the amplitude of the LPC was larger in the inclusion by others category than in both the friend (*p* = 0.011) and stranger (*p* < 0.001) exclusion categories. These results suggest that social exclusion triggers a state of heightened alertness and impairs inhibitory control, regardless of the source of the relationship. This is evidenced by the lack of significant differences in N270 and LPC amplitudes between friend and stranger exclusion. These results suggest that while the cognitive control failure theory provides a reasonable explanation for certain aspects of the data, it may not fully account for the observed phenomena. By contrast, the relevant theory of social exclusion—which emphasizes both resources and motivation—provides a better explanation for these phenomena. This study contributes to understanding the inhibitory control mechanisms underlying behavioral responses after social exclusion, and the findings further support the value of theories that emphasize both resources and motivation when interpreting such responses.

## 1. Introduction

Social exclusion refers to the process by which an individual’s need for belonging and relationships is hindered by rejection or exclusion from a social group or its members (Williams, 2007; Büttner & Rudert, 2022). This exclusion has been shown to trigger aggressive and impulsive behaviors (Leary et al., 2006), which is a finding consistently supported by recent research. For example, Andrighetto et al. (2019) observed heightened hostility among male participants who experienced rejection from their ideal romantic partners. Similarly, Gabbiadini and Riva (2018) found that excluded individuals demonstrated a stronger preference for playing violent games over nonviolent or prosocial alternatives.

Current theories explain how behavioral responses to social exclusion primarily center on the Temporal Need–Threat Model, the Cognitive Control Failure Theory, and the Multi-Motivation Model. The Temporal Need–Threat Model (Williams, 2007; Ren et al., 2018) suggests that individuals undergo three stages after exclusion: the reflexive stage, the reflective stage, and the resignation stage. The Cognitive Control Failure Theory conceptualizes the experience of exclusion as analogous to being struck by a blunt weapon—an impact that triggers a state of cognitive disintegration and impairs cognitive control. As a result, individuals are left with insufficient resources to regulate their behavior, often leading to impulsive actions (Baumeister et al., 2005; Twenge et al., 2001, 2003; Otten & Jonas, 2013). The Multi-Motivation Model, in contrast, emphasizes that while initial responses to exclusion are relatively uniform, subsequent behaviors vary significantly depending on individuals’ motivations, interpretations, and appraisals of the exclusion event (Smart Richman & Leary, 2009).

Inhibitory control refers to the ability to suppress inappropriate behaviors, thoughts, or emotions (Diamond, 2013; Gratton et al., 2018). It is closely related to broader constructs such as executive function and cognitive control, which are also widely used in research contexts. During inhibitory control tasks, social exclusion has been shown to increase error rates (Peng et al., 2018). For example, in a study using an anti-saccade task to assess the effect of exclusion on inhibitory control, participants who experienced social exclusion made significantly more errors than those in the included group (Jamieson et al., 2010). Additionally, Lurquin et al. (2014) used the “end-up-alone” paradigm to induce social exclusion and combined it with ERP recordings. They found that excluded individuals exhibited larger N450 amplitudes during the Stroop task under consistent (compared to inconsistent) conditions relative to both inclusion and control groups. In a study employing the Cyberball paradigm to induce social exclusion, the results demonstrated that excluded individuals exhibited impaired inhibitory control during a visual search task, as reflected by reduced Pd amplitudes (Xu et al., 2017). Xu et al. (2016) employed the Cyberball paradigm alongside a Go/Nogo task and event-related potential (ERP) measurements to investigate the impact of social exclusion by strangers on cognitive control. Their findings indicated that, in terms of conscious cognitive control, excluded individuals displayed larger N2 amplitudes and smaller P3 amplitudes compared to included individuals. These results suggest that although excluded individuals allocate greater attention to conflict detection, they exhibit reduced investment in the conscious inhibition of impulsive responses. This pattern indicates that social exclusion simultaneously enhances sensitivity to conflict monitoring while impairing inhibitory control. Similarly, a study employing the Cyberball paradigm demonstrated that social exclusion leads individuals to allocate greater cognitive resources to conflict detection—reflected in larger N2 amplitudes—while reducing resources devoted to behavioral inhibition, as indicated by diminished P3 amplitudes (Otten & Jonas, 2013).

Previous studies have not only scrutinized the direct impact of social exclusion on inhibitory control but have also delved into the mediating role of inhibitory control in subsequent behaviors. Cognitive Control Failure Theory postulates that social exclusion undermines an individual’s capacity for inhibitory control, initiating a cascade of detrimental effects. For instance, research has demonstrated a negative correlation between impulsive behavior displayed by excluded individuals and their cognitive abilities (Wilkowski et al., 2010; Will et al., 2016). In a study by Hames et al. (2018), the end-up-alone paradigm was utilized to induce social exclusion, with participants subsequently categorized into a future loneliness group, a future belonging group, and a no-feedback control group. The findings revealed that, following social exclusion, the future loneliness group demonstrated heightened social pain, diminished executive function, increased suicidal ideation, and a strong positive correlation with self-harm behavior. Research further demonstrates that the experience of social pain, along with its associated negative emotions, can propel excluded individuals toward seeking control over others. This frequently leads to increased aggression and antisocial acts, which function as a compensatory pathway for achieving a sense of control and existential validation (Kawamoto et al., 2015). Additionally, socially excluded individuals with attachment anxiety experience a greater loss of executive control when facing unjust punishment. This impairs their ability to allocate cognitive resources to suppress aggressive impulses during evaluation and decision-making (DeWall et al., 2011; Watkins et al., 2015), thereby increasing reactive aggression.

Research on social exclusion and inhibitory control reveals a remarkably consistent pattern: irrespective of the specific paradigm used to induce feelings of exclusion, a core feature of these studies is the comparison between a group experiencing social exclusion and a control group experiencing social inclusion. Moreover, the existing literature in this area has predominantly examined exclusion enacted by strangers. However, Nezlek et al. (2012) revealed a wider spectrum of exclusion sources, noting that while 32% of instances stem from strangers, a significant majority originate from other relationships, including unfamiliar individuals (30%), casual friends (16%), close friends (13%), romantic partners (4%), and relatives (5%). This indicates that both close and distant relationships can be sources of exclusion; however, research has largely overlooked how the degree of interpersonal closeness influences the experience and outcomes of being excluded. Thus, leveraging the framework of relational distance, the current study aims to determine how the source of exclusion (e.g., friend vs. stranger) influences subsequent inhibitory control performance. Furthermore, in line with the Multiple Motivation Model (Smart Richman & Leary, 2009), it is hypothesized that exclusion from close versus distant sources will elicit different patterns of behavioral responses. For instance, if individuals perceive a relationship with an excluder as highly valuable (e.g., a close friend), they may attempt to repair it by engaging in prosocial behavior. Conversely, they may exhibit antisocial tendencies or social withdrawal when the relationship holds low relational value. This theoretical prediction finds empirical support in the work of Uskul and Over (2014), who demonstrated that farmers and pastoralists exhibited more avoidance and less friendly behavior when excluded by strangers compared to close others. This study investigates how social exclusion from varying relational distances influences inhibitory control, thereby exploring the cognitive mechanisms underlying these behavioral differences. By doing so, it will contribute to a clearer understanding of how different relational contexts shape individuals’ cognitive and behavioral responses to social exclusion.

To investigate the research question, this study will adopt the following approach. Firstly, friends will be selected to represent close relationships, and strangers will be used to represent distant relationships. Furthermore, a control group designated as “others inclusion” will be included in the study. This condition is characterized by a social environment in which the experimental participant is fully accepted by all others. Secondly, drawing on the methodology of Ikeda and Takeda (2019), the static passing ball paradigm will be used to manipulate social exclusion and inclusion. To ensure uniformity of visual stimuli across all three conditions, the color-identity association paradigm will be introduced. Under this framework, virtual avatars representing different social identities will be assigned the same color, thereby controlling for extraneous visual variation while maintaining distinct identity cues. Finally, the Go/Nogo paradigm is an experimental task used to assess response inhibition. Participants must respond to common “Go” stimuli (e.g., by pressing a button) and resist responding to rare “Nogo” stimuli. Based on this description, it is evident that this paradigm is highly suitable for our research. Therefore, we combined the Go/Nogo task with event-related potential (ERP) measurements to evaluate how being excluded by individuals with varying levels of intimacy affects inhibitory control.

## 2. Materials and Methods

### 2.1. Participants

A total of 95 college students (42 males) were recruited via campus advertisements and randomly assigned to one of three experimental groups. Following the experiment, data from seven participants were excluded: four due to misunderstanding the instructions and consequently making excessive keystroke errors, and three due to poor-quality EEG recordings. Finally, the final sample consisted of 29 participants in the inclusion by others group (M_age_ = 19.48 ± 0.87 years; 13 males), 31 in the friend-exclusion group (M_age_ = 19.10 ± 0.83 years; 14 males), and 28 in the stranger-exclusion group (M_age_ = 19.14 ± 0.75 years; 13 males). The sample size was determined using G*Power 3.1 software. Based on an alpha level of 0.05, a statistical power of 0.80, and a moderate effect size (*ƒ* = 0.25), the required sample size was estimated to be 81 participants. Thus, the final sample in this study is sufficient to achieve adequate statistical power. All participants were right-handed, in good health, free from color blindness or color weakness, and had normal or corrected-to-normal vision. Prior to the experiment, each participant was asked to provide basic demographic information, read through, and sign the informed consent form. Upon completion of the experiment, they received monetary compensation. This study was approved by the Academic Ethics Committee at the corresponding author’s university.

### 2.2. Study Design

This experiment employed a 3 (social priming: others inclusion, friend exclusion, stranger exclusion) × 2 (stimulus type: Go, Nogo) mixed-design ANOVA. The social priming condition was manipulated as a between-subjects variable, while the stimulus type was treated as a within-subjects variable. The dependent variables included participants’ response time, accuracy, and the corresponding ERP components.

### 2.3. Experimental Procedures and Materials

Firstly, participants completed the Inclusion of Others in the Self (IOS) Scale to assess their perceived closeness with both friends and strangers. The experiment then proceeded in two phases. The first phase consisted of two steps: (1) a color-identity association task, in which participants learned to link specific-colored figurines to different identities, and (2) a social exclusion/inclusion priming task. Both procedures in this phase followed the protocol established in our team’s previously published work (Zhang et al., 2023). The second phase involved an inhibitory control experiment.

Stage 1, Step 1: Color-identity connection task

**Figure 1:**
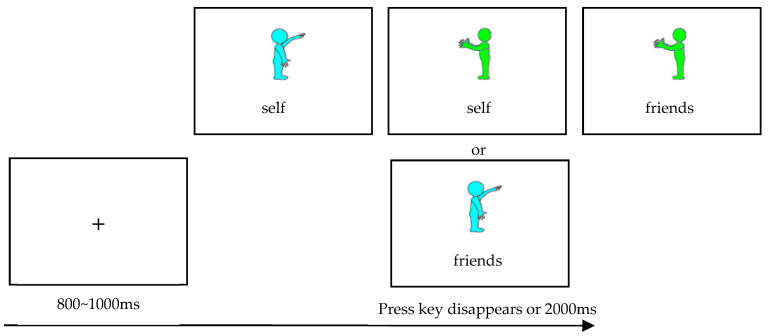
Schematic diagram of the color-identity connection task experiment process.

In this task, participants were provided with different colored figurines (as shown in Figure 1) to symbolize various types of interpersonal relationships—either close or distant—according to specific experimental instructions. The study employed a between-groups design in which cyan figurines were assigned to represent friends, strangers, or others depending on the experimental condition, while green figurines consistently represented the participants themselves. To mitigate the potential influence of color-related associations, the assignment of colors to relationship types was systematically counterbalanced across participants. The detailed procedure for each trial (depicted in Figure 1) consisted of the following steps: ① A fixation cross (“+”) appeared at the center of the screen to cue participants to focus their attention. ② Simultaneously, figurines of different colors along with corresponding identity labels were presented. Participants were instructed to judge whether the color of the figurine matched the identity label. ③ If they perceived a match, they pressed the “F” key; otherwise, they pressed the “J” key. Key assignments were counterbalanced across participants.

Initially, participants completed 12 practice trials (three trials per condition, as shown in Figure 1) to familiarize themselves with the experimental task and procedure. They advanced to the formal experiment only after achieving an accuracy rate exceeding 95%. The trial structure in the formal experiment was identical to that of the practice phase. Each condition was presented 12 times, resulting in a total of 48 trials. The effectiveness of this method in establishing strong associations between colored figurines and identities has been validated in multiple studies (Golubickis et al., 2020; Yin et al., 2019).

Stage 1, Step 2: Passing ball task

**Figure 2:**
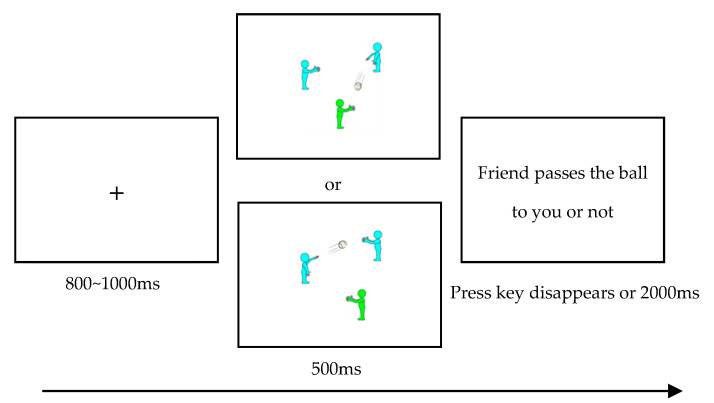
Schematic diagram of the experimental process of the passing ball task.

Drawing on methods adapted from prior studies (Ikeda & Takeda, 2019; Zadro et al., 2004), participants were informed that the experiment aimed to improve mental visualization skills through game-based tasks. They were instructed to actively engage their mental imagery in accordance with the scenarios presented. Throughout the session, static images were used to simulate dynamic ball-passing interaction. Throughout the process, static images were used to simulate a dynamic ball-passing interaction. Participants were asked to vividly imagine the scenario and judge whether the friend or the stranger passed the ball to them. For instance, when presented with the image in the upper part of Figure 2, participants were asked to imagine themselves participating in the game, visualizing their intention to catch the ball and successfully catching it. When shown the image in the lower part of Figure 2, participants were instructed to engage in the game while imagining their intention to catch the ball but visualizing that the other player did not pass the ball to them.

The detailed procedure for each trial (illustrated in Figure 2) consisted of the following steps: ① A fixation cross (“+”) was presented at the center of the screen to prompt attention focus. ② Static images representing ball-passing scenarios were then displayed in random order. During this phase, no key press response was required; instead, participants were instructed to engage in vivid mental imagery based on the depicted scenario. ③ Finally, a question appeared asking: “friend/stranger/others pass the ball to you or not”. Participants indicated their response by pressing the “D” key for “no” and the “K” key for “yes”. Key assignments were counterbalanced across participants.

Initially, participants completed 16 practice trials—comprising eight trials in which the ball was passed to them and eight in which it was not—to familiarize themselves with the experimental task and procedure. They proceeded to the formal experiment only after achieving an accuracy rate of 95% or higher. The formal experiment followed the same procedure as the practice phase and consisted of 80 trials. For the two social exclusion groups, exclusion was induced by presenting trials in which the ball was passed to the participant in only 20% of the cases, while not passed in the remaining 80%. In contrast, the inclusion group experienced social inclusion through an equal distribution of passes and non-passes, with each outcome presented in 50% of the trials.

Stage 2: Inhibition control experiment

**Figure 3:**
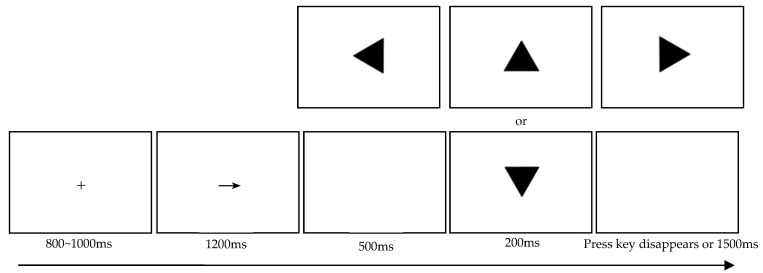
Schematic diagram of the Go/Nogo task.

The specific experimental procedure (see Figure 3) was as follows: ① A fixation cross (“+”) was displayed at the center of the screen. ② An arrow pointing either left (“←”) or right (“→”) was then presented. ③ Following a blank screen, triangles with different orientations appeared. Participants were instructed to respond according to the arrow direction and stimulus type: When a left-pointing arrow was shown, they were to press the “F” key for Go stimuli and withhold response for No-go stimuli. When a right-pointing arrow appeared, they were to press the “J” key for Go stimuli and withhold response for No-go stimuli. As illustrated in Figure 3, the triangle with a vertex pointing directly downward was designated as the No-go stimulus, while all other orientations served as the Go stimulus.

Participants first completed 32 practice trials (24 Go trials and 8 No-go trials) to become familiar with the experimental task and procedure. They proceeded to the formal experiment only after achieving an accuracy rate of 95% or higher. The procedure in the formal experiment was identical to that of the practice phase. A total of 192 trials were administered, with Go and No-go conditions presented in a 3:1 ratio—specifically, 144 Go trials and 48 No-go trials. All trials were presented in random order.

### 2.4. EEG Data Recording and Analysis

Electroencephalogram (EEG) data were recorded using a 64-channel system (Anti Champ model, Brian Product Company) with electrodes arranged according to the international 10–20 system. Horizontal electrooculogram (EOG) signals were monitored via electrodes placed 1 cm laterally to the external canthus of the right eye, and vertical EOG was recorded using electrodes positioned 1.5 cm below the lower eyelid of the left eye. A ground electrode was attached to the forehead, and the Fz electrode served as the reference during recording. The EEG signal was sampled at 500 Hz with an online bandpass filter of 0.01–100 Hz. All electrode impedances were maintained below 5 kΩ. Offline EEG analysis was performed using Brain Vision Analyzer 2.2. The data processing pipeline consisted of the following steps: Re-referencing: The raw data were re-referenced offline to the average of the electrodes placed on the bilateral mastoids. Filtering: A zero-phase shift filter was applied with a bandpass of 0.1–30 Hz to remove low-frequency drift and high-frequency noise, including 50 Hz power line interference. Ocular Artifact Correction: Independent Component Analysis (ICA) was conducted to identify and remove components associated with eye movements and blinks. Epoching: The continuous data were segmented into 1000-ms epochs time-locked to stimulus onset, including a 200-ms pre-stimulus baseline and an 800-ms post-stimulus interval. Baseline Correction and Artifact Rejection: After baseline correction, epochs containing amplitudes exceeding ±80 μV or with voltage steps greater than 50 μV/ms were excluded from further analysis. Averaging: Epochs were averaged separately for each of the three experimental conditions. Subsequently, based on the grand-average ERP waveforms and in line with previous research (Cheng et al., 2019; Rivera & Soylu, 2021; Wang et al., 2000), the N270 (240–300 ms, a negative wave emerging 270 ms post-stimulus) and LPC (300–450 ms, Late Positive Component, emerging 300–600 ms post-stimulus) components over frontal regions (electrodes F1, Fz, F2) were selected for statistical analysis. All analyses were performed using SPSS 19.0. To control for Type I error, p-values from post hoc multiple comparisons following ANOVA were corrected using the Bonferroni method.

## 3. Results

### 3.1. IOS Measurement Results

A between-subjects ANOVA was conducted to examine the effect of the social priming group on intimacy, as measured by the IOS, with friends and strangers. The results indicated no significant differences in intimacy ratings among the three participant groups (all *Fs* < 0.47, *ps* > 0.628). Additionally, related samples t-tests were performed to compare intimacy ratings between friends and strangers within each group. These analyses revealed significant differences in all three groups, with consistently higher intimacy ratings for friends compared to strangers (all *ts* > 12.17, *ps* < 0.001). Together, these findings demonstrate a clear and robust distinction in perceived intimacy between friends and strangers across all experimental conditions.

### 3.2. Behavioral and ERP Results

A repeated measures ANOVA of 3 (social priming group: others inclusion, friend exclusion, stranger exclusion) × 2 (stimulus type: Go, Nogo) was performed on the correct rate and response time and found that: On the correct rate, the main effect of social priming group was not significant *F*(2, 85) = 1.03, *p* = 0.361, the main effect of stimulus type was significant *F*(1, 85) = 29.38, *p* < 0.001, $\eta_{p}^{2}$ = 0.257, and the interaction between them was not significant *F*(2, 85) = 0.81, *p* = 0.450. In response time, the main effect of social priming group was not significant *F*(2, 85) = 0.62, *p* = 0.540, the main effect of stimulus type was significant *F*(1, 85) = 53.53, *p* < 0.001, $\eta_{p}^{2}$ = 0.386, and the interaction between them was not significant *F*(2, 85) = 1.14, *p* = 0.323.

N270. Repeated-measures ANOVA of 3 (social priming group: others inclusion, friend exclusion, stranger exclusion) × 2 (stimulus type: Go, Nogo) on the amplitudes of the N270 components in the frontal region evoked by the Go/Nogo task found that: The main effect of social priming group was marginally significant *F*(2, 85) = 6.06, *p* = 0.003, $\eta_{p}^{2}$ = 0.125, the main effect of stimulus type was not significant *F*(1, 85) = 1.39, *p* = 0.242, and the interaction between them was not significant *F*(2, 85) = 1.21, *p* = 0.304. Combining the post hoc comparison and descriptive results, it can be seen that under the Go(other inclusion: 2.06 μV, friend exclusion: 0.26 μV, stranger exclusion: −1.26 μV) and Nogo(others inclusion: 2.95 μV, friend exclusion: −0.06 μV, stranger exclusion: −0.63 μV) conditions, the amplitude of N270 components induced by stranger exclusion (*p* = 0.001), and friend exclusion (*p* = 0.018) was larger than that induced by others inclusion.

**Figure 4:**
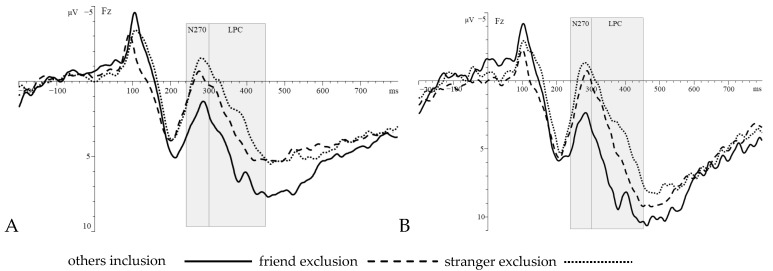
The ERP waveforms of the three social priming groups under the conditions of Go (**A**) and Nogo (**B**).

LPC. Repeated-measures ANOVA of 3 (social priming group: inclusion by others, friend exclusion, stranger exclusion) × 2 (stimulus type: Go, Nogo) on the amplitudes of the LPC components in the frontal region evoked by the Go/Nogo task found that: The main effect of social priming group was significant *F*(2, 85) = 7.74, *p* = 0.001, $\eta_{p}^{2}$ = 0.154, the main effect of stimulus type was significant *F*(1, 85) = 13.91, *p* < 0.001, $\eta_{p}^{2}$ = 0.141, and the interaction between them was not significant *F*(2, 85) = 0.94, *p* = 0.393. Combining the post hoc comparison and descriptive results, it can be seen that under the conditions of Go(others inclusion: 4.88 μV, friend exclusion: 2.87 μV, stranger exclusion: 1.41 μV) and Nogo (others inclusion: 7.19 μV, friend exclusion: 3.94 μV, stranger exclusion: 2.60 μV) conditions, the amplitude of LPC components induced by others inclusion was larger than that induced by friend exclusion (*p* = 0.011) and stranger exclusion (*p* < 0.001) (Figure 4).

## 4. Discussion

This study employed a bimanual Go/Nogo task combined with event-related potential (ERP) technology to examine how exclusion based on close versus distant relationships affects individual inhibitory control. As noted by Otten and Jonas (2013), opposing ERP components (N270 and LPC) may neutralize each other’s effects, potentially resulting in no significant differences in behavioral performance between exclusion and inclusion groups. The present findings are consistent with this earlier observation. Thus, within the Go/Nogo paradigm, greater emphasis should be placed on EEG-derived outcomes. The ERP results revealed that in both Go and Nogo conditions, friend-exclusion and stranger-exclusion elicited larger N270 amplitudes compared to inclusion by others. Previous studies have indicated that the human brain possesses a conflict monitoring system. When an individual processes conflicting information, an N270 component is elicited over frontal regions (Rivera & Soylu, 2021; Ma et al., 2021; Zhao et al., 2020; Wang et al., 2000). The larger amplitude of this component reflects greater allocation of attention and heightened alertness to conflicting information. Thus, the present findings suggest that when individuals experience exclusion, they allocate greater attention to task-relevant conflicting information and exhibit increased alertness during inhibitory control tasks. According to the resource-limited theory, attentional resources throughout the cognitive control process are finite and shared (Kahneman, 1973). This perspective suggests that because the inclusion by others group exhibited lower alertness and consumed fewer cognitive resources during the early stage of the inhibitory task, more attention-related resources remained available for later processing stages. Consequently, it can be inferred that the inclusion by others group should elicit larger LPC amplitude compared to the exclusion groups. The results of this study align with the aforementioned inference and are consistent with previous research. Prior studies have demonstrated that, compared to social inclusion, the threat to basic needs and the social pain resulting from exclusion diminish the cognitive resources available to individuals. This resource depletion leads to insufficient capacity for subsequent cognitive control tasks. Specifically, this is reflected in reduced amplitudes of late ERP components (such as LPC and P3) during tasks requiring inhibitory control, like the Nogo task (Xu et al., 2016; Otten & Jonas, 2013).

Regulatory Focus Theory provides a reasonable explanatory framework for the aforementioned research findings. According to Regulatory Focus Theory (Higgins, 1997; Molden et al., 2009; Park & Baumeister, 2015), individuals adopt two distinct motivational orientations when pursuing specific goals—such as performing a conflict task in an experimental context. These include a promotion focus, centered on advancement needs (advancement, i.e., growth, development, training, etc.), and a prevention focus, oriented toward safety needs (security, i.e., protection, invulnerability, etc.). The two regulatory foci exhibit distinct emotional and behavioral patterns during goal pursuit. Individuals with a prevention focus are more attentive to potential negative outcomes, experience emotions related to relief and anger, demonstrate heightened vigilance toward threats, and exhibit a tendency to adopt safety-oriented responses. In contrast, individuals with a promotion focus are more concerned with the presence of positive outcomes, experience emotions associated with joy and disappointment, are more inclined to make reward-seeking decisions, and generally strive to achieve positive results. Research has shown that individuals exhibit a stronger prevention focus when socially excluded, whereas a stronger promotion focus is observed following social inclusion (Molden et al., 2009; Park & Baumeister, 2015). It can be inferred that individuals in the exclusion groups, influenced by a prevention-focused motivation, may perceive the presented conflicting information as a potential threat, thereby exhibiting heightened alertness. Specifically, for individuals in exclusion groups, elevated alertness to potential threats diverted extensive cognitive resources to early conflict monitoring (evidenced by increased N270 amplitude), thereby compromising later resource availability for task-relevant processing, as manifested by a reduced LPC amplitude. In contrast, individuals in the inclusion group, who exhibit lower threat vigilance, allocate fewer cognitive resources during early processing (as indicated by a smaller N270 amplitude), thereby reserving more resources for later task completion, which is reflected in a larger LPC amplitude.

As described in the previous section, motivational orientation can play a regulatory role in cognitive resource allocation. A review of previous literature indicates that the resource-limited cognitive control failure theory is frequently employed to explain the psychological mechanisms underlying negative behaviors following social exclusion. According to this view, the emergence of negative behaviors toward others or oneself following exclusion stems from a reduction in available cognitive resources, which impairs cognitive control and thereby undermines the ability to suppress various negative behaviors (Baumeister et al., 2005; Leary et al., 2006; Lurquin et al., 2014). While this theoretical model demonstrates considerable explanatory power in dichotomous comparisons involving only exclusion and inclusion groups, its applicability appears limited in studies incorporating multiple types of exclusions. The findings of the present study, which included two distinct exclusion groups, suggest that the explanatory pathway described above exhibits important limitations. Previous research has demonstrated that individuals employ significantly different coping behaviors in response to exclusion by close versus distant others. For example, people exhibit more avoidance behavior when excluded by strangers compared to exclusion by close others (Uskul & Over, 2014). If the differences in coping behaviors were solely due to impaired cognitive control, as the aforementioned account suggests, then significant differences in cognitive control outcomes between the two exclusion groups would be expected. However, although some variation in the decline of cognitive control was observed between the two exclusion groups in this study, these differences were not statistically significant. These findings further indicate that the aforementioned explanatory framework offers only a partial account of the behavioral changes observed following social exclusion, rather than a complete causal explanation. It can therefore be concluded that diminished cognitive control contributes to, but does not fully determine, the emergence of negative behaviors after exclusion. Additional determinants of negative behaviors may be more reasonably explained by considering underlying motivational mechanisms. For example, the reasons for adopting more negative behaviors in response to the exclusion of strangers are, on the one hand, the decline in cognitive control due to exclusion, and on the other hand, individuals do not think that strangers are very important to them, so they have no motivation to repair the relationship. The convergence of these cognitive and motivational factors elicits stronger negative behavioral responses to strangers than to close others. Therefore, a social exclusion theory that integrates both resource and motivational perspectives offers a more comprehensive and plausible explanation for the psychological mechanisms underlying the diverse coping behaviors individuals exhibit following exclusion. Future research in the field of social exclusion should aim to integrate cognitive control failure theory with a multimotive model, thereby developing a more comprehensive theoretical framework to better explain individual behavioral responses. While this study has yielded valuable findings, it also has several limitations. As part of our research team’s series of studies, we continued to use the static passing ball task to induce social exclusion. Although its induction effect has been empirically validated, how it compares to dynamic tasks requires further investigation. The experiment primarily observed differences at the neurophysiological level, with no significant behavioral differences detected. Future studies should optimize experimental design and paradigms to achieve more comprehensive results. In terms of interpersonal relationships, the study only distinguished between friends and strangers, and the participants were primarily drawn from young Chinese university students. Future research should further refine the categorization of relationship closeness and expand the participant pool to verify the robustness and generalizability of the findings.

## 5. Conclusions

This study employed a bimanual Go/Nogo paradigm combined with event-related potential (ERP) technology to examine how exclusion based on close versus distant relationships affects individual inhibitory control. The results revealed that both friend exclusion and stranger exclusion elicited enhanced N270 amplitudes in frontal regions compared to the inclusion group. Conversely, inclusion evoked larger LPC amplitudes than either exclusion group. These findings suggest that social exclusion heightens alertness and impairs inhibitory control; however, the moderating role of relationship type (close vs. distant) was not significant.

## Data Availability

Data are available from the corresponding author upon reasonable request.
